# Conservation of ancient full-length open reading frames in vertebrate endogenous retroviruses

**DOI:** 10.1186/1742-4690-8-S2-P47

**Published:** 2011-10-03

**Authors:** Hugo Martins, Palle Villesen

**Affiliations:** 1 Bioinformatics Research Centre, Aarhus University, Denmark; 2 PhD Program in Computational Biology, Instituto Gulbenkian de Ciências, Oeiras, Portugal

## Background

Endogenous retroviruses (ERVs) are genetic remnants of exogenous retroviral infections that have endured selective processes and made their way into ancestral host genomes. While many of them have deteriorated beyond the ability to code for detectable proteins, some still retain activity or the potential for coding activity [1-3]. A few active ERVs have been coopted into fully functional genes that play important roles in development or disease [1, 2, 4]. New high throughput data revealed that these ERVs are far more common than previously thought and different software packages have been created to search genomes for these structures. In this study, we build on such a tool to look at several vertebrate genomes, search for ERV remnants and identify loci with long, intact open reading frames for the major retroviral genes and dating these loci of interest using the long terminal repeat (LTR) divergence method [5].

## Materials and methods

The genomes of twelve vertebrates were acquired from UCSC Genome Browser and analyzed with an in-house method based on the *Itrharvest*[*6*] software package, complemented with additional filtering and data retrieval. Candidate ERV loci were then probed for full-length open reading frames, which were classified within one of the three major retroviral genes: *gag*, *pol* or *env* by a BlastX [7] against a retroviral protein database. LTR dating for each ERV locus was done by comparing the genetic divergence between the LTRs.

## Results/Conclusions

Our study reveals the existence of a few conserved full length open reading frames in loci whose LTRs present a similarity lower than 70% and plenty of open reading frames occur across all genomes in loci with LTR similarity below 80%. Current method limitations may cause this number to be an underestimation but nonetheless reveals the existence of old retroviral infections that have, to this date, kept some coding potential. In the studied primate genomes, a select number of loci still retain long to full length open reading frames on all three major genes even on loci with LTR divergence under 95% (Table 1). *Pol* genes appear to possess the highest number of conserved ORF and a high number of recent integrations with full conservation has been found in the mouse genome.

**Table 1:** **Number of ERV loci containing large intact open reading frames (at least 1000 uninterrupted nucleotides) for all three major retroviral genes:** ***gag*****,** ***pol and env*****,grouped by their locus'LTR divergence percentage**

	<**80** %	80-85%	85-90%	90-95%	>**95** %	total
Human	0	0	0	1	8	10
Chimpanzee	0	0	0	0	3	3
Orangutan	0	0	0	1	0	1
Macaque	0	0	0	0	2	2
Dog	0	0	0	0	1	1
Mouse	0	0	0	3	169	172
Cow	0	0	0	1	7	8
Opossum	0	0	0	7	33	40
Chicken	0	0	0	0	2	2
